# Polymorphisms in vitamin D receptor, toll-like receptor 2 and Toll-Like receptor 4 genes links with Dengue susceptibility 

**DOI:** 10.6026/97320630017506

**Published:** 2021-04-30

**Authors:** Arvind Kumar Singh, Shantanu Prakash, RK Garg, Parul Jain, Rashmi Kumar, Amita Jain

**Affiliations:** 1 Department of Microbiology King George's Medical University, Lucknow, UP, India; 2 Department of Neurology King George's Medical University, Lucknow, UP, India; 3 Department of Paediatrics King George's Medical University, Lucknow, UP, India

**Keywords:** Innate Immunity, Dengue virus Infection, SNP, Toll like Receptor, TLR 2, TLR4, Vitamin D receptor (VDR)

## Abstract

Host genetic factors are known to determine disease susceptibility in dengue virus infection. Therefore, in this study association of gene polymorphisms of Vitamin D Receptor [rs731236 (Taq) and rs7975232 (Apa1)], Toll-like receptor 2 [rs5743708 (Arg735Gln) and rs5743704 (Pro631His)] and Toll-like receptor 4 [rs4986790A/G(Asp299Gly13843) and rs4986791 C/T(Thr399Ile)] were studied in cases with dengue as compared to controls. Total 98 cases of confirmed dengue virus infection and 98 age, sex and geographically matched healthy controls were enrolled and their genetic polymorphisms for the above mentioned regions were studied by Sanger sequencing. Mutant genotypes CC of VDR rs731236 (Taq1) [(OR 3.808, p value =0.02, CI 1.160-12.498)], GG of VDR rs7975232 (Apa1) [(OR 3.485, p value =0.02, CI 1.162-10.45)] and heterozygous genotypes of TLR4 rs4986790 A/G Asp299Gly [OR 2.40, p value= 0.02, CI 1.12-5.14], TLR4 rs4986791 C/T Thr399Ile [OR 2.09, p value=0.02, CI 1.12-5.14] were found to be significantly more in cases with dengue virus infection as compared to the controls. Also, at these positions mutant alleles were observed in significantly higher number of cases than controls. The values for C allele at VDR rs731236 (Taq1) were OR 1.86, p value 0.009, CI 1.162-3.001; for allele G at rs7975232( Apa1) were OR 2.71, p value 0.006, CI 1.196-2.98 for allele G at TLR4s rs4986790 A/G Asp299Gly were OR 2.35, p value 0.009, CI 1.23-4.50 and for allele T at rs4986791 C/T Thr399Ile were OR 2.36, p value=0.006, CI 1.28-4.38. VDR and TLR4 but not TLR2 gene polymorphisms were found to be associated with dengue susceptibility in Indian population.

## Background:

Dengue fever is one of the most important arthropod borne endemic diseases in some Asian and Latin American countries [1]. The host susceptibility to dengue may be influenced by host immune response and genetic factors, which includes single nucleotide polymorphisms (SNPs) of genes such as Toll like receptors 2 and 4 (TLR-2, TLR-4) present on the cytoplasmic membrane [2] and Vitamin D Receptor (VDR) present on nuclear membrane [3]. Multiple important roles of VDR are known such as modulation of immunoregulatory effects of 1,25 dihydroxy vitamin D3, monocyte activation, stimulation of cellular immune responses, antagonism production of immunoglobulin and regulating lymphocyte proliferation [3]. Therefore it may also affect pathogenesis of Dengue virus infection. TLRs are a family of pathogen recognition receptors (PRRs) that are well known for their role in immune surveillance. PRRs are important as they alert the immune system for the presence of foreign microbes by recognizing and binding to pathogen-associated molecular patterns (PAMPs) [2]. Few functional polymorphisms of TLR2 gene such as Arg735Gln, and Pro 631 His are known, which alter the activity of receptor [4] or impair receptor signalling, which in turn increases the susceptibility to dengue virus infection. TLR4 is present on most immune cells like monocytes and neutrophils. Several SNPs in TLR4, particularly Asp299Gly and Thr399Ile, are known to be associated with severe forms of diseases such as falciparum malaria [2], neurocysticercosis [5]. Role of these polymorphisms in pathogenesis of dengue fever is yet not known. Since several gaps exist in the present knowledge of factors affecting host susceptibility to dengue, the present study was done to study the association between genetic polymorphisms of TLR-2 [rs5743708 (Arg735Gln) and rs5743704 (Pro 631 His)], TLR-4 [A/G (Asp299Gly-13843) and rs4986791 C/T (Thr399Ile)], and VDR [rs731236 (Taq) and rs7975232(Apa1)] with susceptibility to dengue virus infection.

## Materials and methods:

### Study design:

**Figure 1:**
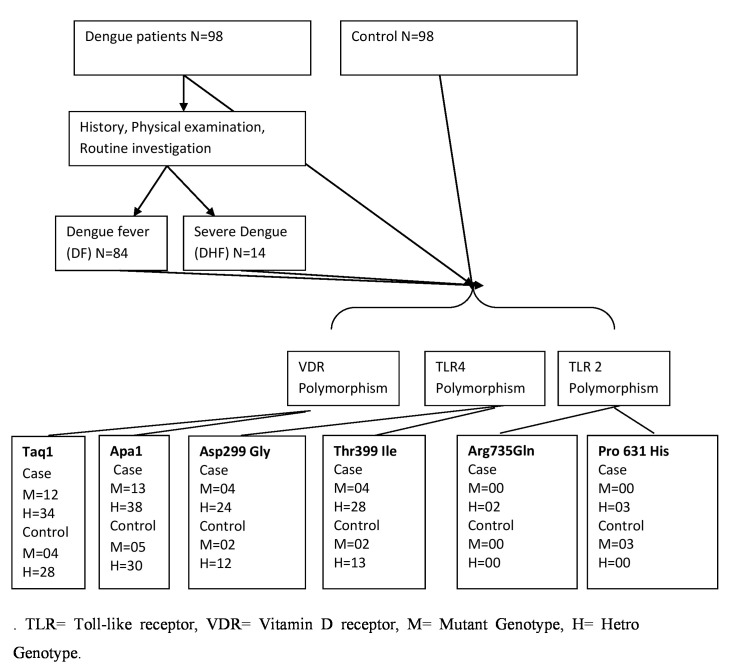
Flow diagram showing the study-design

This prospective case control study was done in Department of Microbiology, in collaboration with the Departments of Neurology and Paediatrics at King George's Medical University (KGMU), Lucknow, India. Ethical approval was obtained from the institutional ethics committee (Registration NO: ECR/262/Inst/UP/2013). Cases and controls were enrolled in the study after obtaining written informed consent. Cases were defined as patient's coming from north India with lab confirmed dengue infection i.e. their serum sample was positive for either anti dengue IgM or DV- non-structural 1 antigen (NS1Ag) or both. Age and sex matched normal healthy volunteers residing in similar geographic area and negative for markers of dengue virus infection were enrolled as controls. Sample size was calculated by QUANTO software; values used were: odds ratio (OR): 2, significance level (α): 0.05, Power of study: 90%, minor allele frequency: 0.2, disease frequency: 18%. Clinical details were recorded in a pre-designed questionnaire and routine blood investigations including complete blood count, liver and renal function test, and electrolytes were measured in all patients. Additionally, cases were classified as severe or non-severe dengue, based on WHO 2009 criteria [5] for further analysis. The flow diagram of study is displayed in (Figure 1).

### Analysis of TLR2, TLR4 and VDR polymorphism:

Whole blood (3 ml) was collected in EDTA vials from cases and controls. Genomic DNA was extracted from whole blood using salting-out method as described elsewhere [6] and was stored at -20°C until tested. TLR2, TLR4 and VDR genotypes were determined by polymerase chain reaction sequencing by chain termination method (sanger-sequencing). The primer sequences were designed in-house (Table 1 - see PDf) and manufactured by Integrated DNA technology, USA. All PCR amplifications were performed in a 25-µL volume of master mix, containing 10X assay buffer, 200 µM each of nucleotide, 1 pm of each primer, 1.0 U of Taq DNA polymerase (Finzymes, Thermo scientific). PCR conditions were as follows: an initial denaturising at 94°C for 10 min, followed by 35 cycles of denaturing at 94°C for 45 sec, annealing at 62.3.6°C (VDR), 55.7°C (TLR-4) & 59.6°C (TLR-2) for 45 sec, extension at 72°C for 1min, final extension at 72°C for 7 min and cooling to 4°C. Template-free water was used as a negative control. After amplification, the products were subjected to exo-sap purification and the purified 1-2 µL PCR products were subjected to bidirectional sequencing PCR utilising BigDye Terminator cycle sequencing Kit v3.1 [7] and single primer. The amplified sequencing PCR products were again purified by ethanol, EDTA and sodium acetate precipitation. The SNPs were analyzed by software BioEdit 7.2.1 sequence alignment editor [8].

### Statistical analysis:

Statistical analysis was done using SPSS version 16.0 (Chicago, LA). All categorical and continuous variables were expressed as percentages and mean ± standard deviation respectively. Chi square test was used to compare categorical variables as applicable. Independent sample T test & Mann-Whitney Test was used to compare means. Binary logistic regression was performed to look for variables independently associated with outcome. A SNP analyzer version 2.0 web-based program was used to determine deviation from Hardy-Weinberg equilibrium. Most frequent homozygous genotype in the control group was used as reference for association analysis. For statistical significance Odds ratios (ORs), p values and corresponding 95 % confidence intervals (95 % CIs) were computed and p<0.05 was considered statistically significant.

## Results:

### Demographic characteristics:

Total 98 cases and 98 controls were enrolled in the study. The mean age of cases and controls was 37.7 and 35.98 years respectively and male to female ratio was 1.72:1 and 1.51:1 respectively. Of the total 98 cases, 84 presented as dengue fever and 14 had severe dengue. Most common symptoms were vomiting (36.9%), body ache (39.28%) and headache (36%). Table 2(see PDF) details the demographic and clinical data of cases and controls.

### Genotypic and allelic distribution:

Table 3 (see PDF) summarizes findings on the genotypic and allelic distributions of SNPs of TLR2 gene, TLR4 gene and VDR gene in all the cases and controls as well as in severe and non-severe dengue cases.

### VDR gene polymorphism:

On genotypic analysis of VDR rs731236 T/C (Taq) polymorphism frequency, mutant genotype (CC) was significantly more common in cases compared to controls (12.2% vs 4.08%, OR =3.80, p value = 0.02, 95% CI=1.16-12.49). The heterozygous genotype (TC) was also more in cases compared to controls but the difference was not statistically significant (34.6% vs 28.5%, OR =1.54, p value = 0.17, CI =0.88-2.86,). The frequency of mutant (C) allele was significantly higher in cases than controls (29.59% vs.18.36%, OR=1.86, p value =0.009CI=1.16-3.001). Likewise, for VDR rs7975232 T/G (Apa1) polymorphism, the frequencies of mutant (GG) and heterozygous (TG) genotypes were significantly more in cases compared to controls, (13.2% vs. 5.1%, OR =3.48, p value = 0.02, CI=1.16-10.45 and 42.4% vs.30.6%, OR 2.04, p value=0.027, CI 1.08-3.84 respectively). The mutant (G) allele was also found more frequently in cases than controls and the difference was significant (32.65% vs. 20.40% OR= 2.72, p value = <0.0064, CI= 1.1961-2.9895) (Table 3 - see PDF).

### TLR 4-gene polymorphism:

On rs4986790 A/G Asp299Gly genotyping analysis heterozygous genotype AG was more significantly observed in cases when compared to controls (24.4% vs 12.04%, OR 2.40, p value 0.02, CI 1.12-5.14). Mutant allele G was also significantly different in cases than controls (16.3% vs 7.6%, OR 2.35, p value 0.009, CI 1.23-4.50) (Table 3 - see PDF). For TLR4 rs4986791 C/T polymorphism, frequency of heterozygous genotype (CT) was significantly more in cases compared to controls (28.5% vs. 13.2%, OR =2.09, p value = 0.037, CI=1.04-4.20,). The difference in mutant (T) allele frequency in cases and controls was statistically significant (36.7% vs. 8.6%, OR= 2.36, p value = 0.006, CI= 1.28-4.38) (Table 3 - see PDF).

### TLR2 gene polymorphism:

No mutant gene was observed in either SNPs of TLR 2 gene i.e. rs5743708 (Arg753Gln) & rs5743704 (Pro631His). In both the SNPs only few heterozygous genotypes were found. No significant difference was observed in genetic polymorphisms between severe and non-severe dengue cases (Table 3 - see PDF).

## Discussion: 

The findings of the present study address the gaps in the existing knowledge about innate immune response and dengue virus pathogenesis, especially in the Indian population. We observed that mutant (CC) and heterozygous (TC) genotypes of VDR rs731236 (Taq1); mutant (GG) and heterozygous (TG) genotypes of VDR rs7975232 (Apa1); mutant (GG) and heterozygous (AG) genotypes of TLR4 rs4986790; and heterozygous (CT) genotypes of TLR4 rs4986791 were significantly associated with dengue fever in our study. Vitamin D receptor (VDR) is an immunomodulator and affects both innate and adaptive immune response whose functioning can be affected by genetic polymorphisms. VDR Taq1 rs731236 and Apa1 rs7975233 SNPs are intronic polymorphisms, which have roles in transcription factor binding and are in linkage disequilibrium with some other functional gene that causes an indirect consequence on disease susceptibility [9]. Known data on the association of VDR polymorphism (Taq1 rs731236 and Apa1 rs7975233) with tubercular meningitis and pulmonary tuberculosis showed association of both polymorphisms with these diseases [10,11]. Associations of the same genetic polymorphisms have also been observed with development of diseases such as chronic hepatitis B virus infection, liver disease progression [12] and acute lower respiratory tract infection (ALRI) [13]. A functional study done in MG-63 human bone derived osteosarcoma cell line demonstrated that presence of mutant haplotype (G-C-T) is associated with 15% lower level of VDR mRNA expression and 30% faster decay of VDR mRNA compared to the wild haplotype (A-A-C) [14]. The findings of our study were in concordance with these studies that showed association of mutant alleles of VDR gene (Taq1 rs731236 and Apa1 rs7975233) with developing dengue virus infection. However, data frp, Maharashtra found significantly lower frequency of mutant allele of Apa1 in all dengue patients as compared to controls [15]. This difference could be due to genetic heterogeneity between the two populations studied. TLR4 are expressed on cell membrane of innate immune cells like monocytes, macrophages, and endothelial cells. It was earlier believed that TLR4 is activated only by the bacterial lipopopysaccharide (LPS) but now it is known that it also mediates immune response by NS1 antigen and other endogenous molecules of dengue virus [16,17] probably by regulation of cytokines like IL-6, TL-12 and interferons that are important in immune response against the virus [18]. Similar to the results of our study, previous researchers have shown that heterozygous genotype of TLR4 Asp299Gly and TLR4Thr399Ile was significantly high in dengue cases than healthy controls [19]. Possible role of TLR4 polymorphism has also been suggested in HIV1 & HIV2 infection and Crimean Congo hemorrhagic fever (CCHF). We could not find any mutant allele in TLR 2 rs5743708 (Arg753Gln) & rs5743704 (Pro631His). Similar results were obtained in studies done on association of same polymorphisms, with TBM and pulmonary tuberculosis [10] although positive association was found in a study from China [20]. This deviation could be due to racial and ethnic factors. The association of these polymorphisms to TLR2 with Dengue virus infection is reported for the first time in this study.

## Conclusion:

In the present study it was observed that VDR gene and TLR 4 gene polymorphisms are associated with dengue virus infection in north Indian population but no such association was observed with TLR2 polymorphism. Expression studies are required further to support these findings.
